# Decellularized fresh homografts for pulmonary valve replacement: a decade of clinical experience^†^

**DOI:** 10.1093/ejcts/ezw050

**Published:** 2016-03-24

**Authors:** Samir Sarikouch, Alexander Horke, Igor Tudorache, Philipp Beerbaum, Mechthild Westhoff-Bleck, Dietmar Boethig, Oleg Repin, Liviu Maniuc, Anatol Ciubotaru, Axel Haverich, Serghei Cebotari

**Affiliations:** a Department for Cardiothoracic, Transplant, and Vascular Surgery, Hannover Medical School, Hannover, Germany; b Department for Pediatric Cardiology and Pediatric Intensive Care, Hannover Medical School, Hannover, Germany; c Department for Cardiology and Angiology, Hannover Medical School, Hannover, Germany; d Cardiac Surgery Center, State Medical and Pharmaceutical University, Chisinau, Moldova

**Keywords:** Congenital heart disease, Pulmonary valve replacement, Tissue engineering, Homograft, Decellularization

## Abstract

**OBJECTIVES:**

Decellularized homografts have shown auspicious early results when used for pulmonary valve replacement (PVR) in congenital heart disease. The first clinical application in children was performed in 2002, initially using pre-seeding with endogenous progenitor cells. Since 2005, only non-seeded, fresh decellularized allografts have been implanted after spontaneous recellularization was observed by several groups.

**METHODS:**

A matched comparison of decellularized fresh pulmonary homografts (DPHs) implanted for PVR with cryopreserved pulmonary homografts (CHs) and bovine jugular vein conduits (BJVs) was conducted. Patients’ age at implantation, the type of congenital malformation, number of previous cardiac operations and number of previous PVRs were considered for matching purposes, using an updated contemporary registry of right ventricular outflow tract conduits (2300 included conduits, >12 000 patient-years).

**RESULTS:**

A total of 131 DPHs were implanted for PVR in the period from January 2005 to September 2015. Of the 131, 38 were implanted within prospective trials on DPH from October 2014 onwards and were therefore not analysed within this study. A total of 93 DPH patients (58 males, 35 females) formed the study cohort and were matched to 93 CH and 93 BJV patients. The mean age at DPH implantation was 15.8 ± 10.21 years (CH 15.9 ± 10.4, BJV 15.6 ± 9.9) and the mean DPH diameter was 23.9 mm (CH 23.3 ± 3.6, BJV 19.9 ± 2.9). There was 100% follow-up for DPH, including 905 examinations with a mean follow-up of 4.59 ± 2.76 years (CH 7.4 ± 5.8, BJV 6.4 ± 3.8), amounting to 427.27 patient-years in total (CH 678.3, BJV 553.0). Tetralogy-of-Fallot was the most frequent malformation (DPH 50.5%, CH 54.8%, BJV 68.8%). At 10 years, the rate of freedom of explantation was 100% for DPH, 84.2% for CH (*P* = 0.01) and 84.3% for BJV (*P*= 0.01); the rate of freedom from explantation and peak trans-conduit gradient ≥50 mmHg was 86% for DPH, 64% for CH (n.s.) and 49% for BJV (*P* < 0.001); the rate of freedom from infective endocarditis (IE) was 100% for DPH, 97.3 ± 1.9% within the matched CH patients (*P* = 0.2) and 94.3 ± 2.8% for BJV patients (*P* = 0.06). DPH valve annulus diameters converged towards normal Z-values throughout the observation period, in contrast to other valve prostheses (BJV).

**CONCLUSIONS:**

Mid-term results of DPH for PVR confirm earlier results of reduced re-operation rates compared with CH and BJV.

## INTRODUCTION

The use of decellularized valved grafts for pulmonary valve replacement (PVR) was introduced to the clinic at the beginning of this millennium based on the pioneering preclinical work of Shinoka *et al*., who seeded polyglycolic acid fibre matrices *in vitro* with fibroblasts and endothelial cells and implanted the resulting scaffolds as a substitution for pulmonary leaflets in lambs [1].

The possibility of applying artificial tissue-engineered heart valves would solve many unmet clinical demands, such as the permanent availability of different sizes and lengths. These concepts have shown good results in the technical implementation of valved polymeric conduit production and have successfully been used for *in vitro* and *in vivo* seeding of different (stem) cell lines. However, long-term animal models have yet to deliver satisfactory results, due to the lack of mechanical stability of the totally artificial matrices, leading to early failure of valvular function [2].

All tissue-engineering concepts currently in use in the clinic are based on the decellularization of biological scaffolds, such as xenogeneic and allogeneic heart valves, some approaches with and some without pre-seeding with stem cells [3]. Many different protocols for decellularization of biological sources are in use, as well as varying concepts for preservation after processing [4–7].

Haverich *et al*. have developed protocols for the decellularization of donated human heart valves, which avoid the need for cryopreservation and thus minimize risk for the resulting valve scaffold. The first clinical implantations for PVR were performed in 2002, initially with a Trypsin/EDTA decellularization technique and pre-seeding of endothelial progenitor cells in a dynamic bioreactor system. The two paediatric patients were carefully monitored and showed very promising early results [8].

The clinical use of fresh decellularized allografts continued in 2005 using a modified decellularization detergent-based protocol without pre-seeding of stem cells after several groups observed spontaneous recellularization, including recellularization in senescent animal models [9].

In 2011, we published early data on the use of these decellularized fresh pulmonary homografts (DPHs) in children and adolescents in direct comparison with bovine jugular vein conduits (BJVs) and cryopreserved conventional homografts (CHs), the two most widely used PVR alternatives. The findings of this study suggested improved freedom from re-operation and superior haemodynamics using DPHs [10]. The immunological benefit of DPHs was demonstrated in these paediatric patients, who are known to have a fast-reacting immune system, by the lack of activation of the cellular immune system, a leading factor in graft rejection [11]. In a recent study, we analysed the humoral response to DPHs in comparison with decellularized xenogeic and glutaraldehyde-fixed conduits and found significantly fewer specific antibodies against whole-tissue homogenates and the alphaGal epitope [12].

The clinical use of DPHs over the last 4 years has increasingly mirrored a real-life scenario, which was further accelerated by formal approval of DPHs by the competent authorities in 2013 (www.pei.de, ESPOIR PV PEI.G.11634.01.1). Within the present study, we provide an update on the clinical results obtained using fresh, DPHs for PVR, again in comparison with the bovine jugular vein conduits and the cryopreserved conventional homografts.

## MATERIALS AND METHODS

### Study cohort

The present study documents the experiences of two clinical centres with the use of fresh, decellularized pulmonary homografts (DPHs) for PVR. The study was approved by the Medical Ethics Committee of the Ministry of Health in Moldova and by the institutional Ethics Committee of Hannover Medical School, Germany.

Patients from the Cardiac Surgery Center, Chisinau, Moldova and from Hannover Medical School, Hannover, Germany who underwent surgery during the period from January 2005 to September 2015 were enrolled in regular follow-up examinations. All patients were examined, including clinical and functional examinations (echocardiography, ECG, CMR), after surgery, at 6 months and at 12 months, and then every 12 months. Clinical follow-up included a regular physical examination of the patients (physical status, measurements of body height and weight and systemic blood pressure, ECG and New York Heart Association classification). Echocardiographic evaluation (M-mode, two-dimensional, colour flow, pulsatile and continuous-wave Doppler) was performed according the current guidelines of the European Association of Cardiovascular Imaging.

### Surgical procedure

The indication for PVR was confirmed in consultation between paediatric cardiologists and cardiothoracic surgeons in accordance with the current guidelines of the German Society for Pediatric Cardiology (http://www.kinderkardiologie.org/leitlinien/).

Each patient was admitted to the hospital once the appropriate homograft was available and processing completed. Operations were performed under general combined intravenous anaesthesia through a median sternotomy, using a cardiopulmonary bypass with standard bicaval and aortic cannulation and mild hypothermia (32–34°C). Initially, intermittent cold crystalloid or blood cardioplegia was used with a change to beating-heart surgery over the past 4 years. In all cases, the right ventricular outflow tract (RVOT) was reconstructed with an interposition of the DPH, with a continuous suture for the proximal as well as for the distal suture lines. After surgery, aspirin therapy was administered to patients over a period of 3–6 months.

### Conduit degeneration

Degeneration was defined as a maximal peak gradient over any part of the conduit of 50 mmHg or more and/or at least moderate regurgitation (0, none; 0.5, trace; 1.0, mild; 1.5, mild to moderate; 2.0, moderate; 2.5, moderate to severe; 3.0, severe regurgitation).

### Statistics

Each DPH patient was matched to one patient who received a BJV (Contegra®) for PVR and to one patient with a conventional cryopreserved homograft (CH). Patients were chosen from an updated contemporary registry of right ventricular outflow tract conduits (RVOT Conduit Registry) with over 2300 conduits included and a total patient follow-up of over 12 000 patient-years, which has been used for contemporary analysis of CH and BJV results [13]. Data within the Registry originate from seven experienced international centres (including Hannover and Chisinau) with a uniform way of data capturing and evaluation.

Patient matching was performed on the basis of the patient's age at implantation, followed by diagnosis and number of previous operations, as well as the number of previous PVRs. A total of 538 BJVs and 694 CHs were identified within the RVOT Conduit Registry, which were available for matching. Age was considered the most critical aspect in growing patients, especially in infants, and less important in adults.

Summaries of the numerical data are given as means and standard deviation. For comparisons of data with skew distribution, we used the Mann–Whitney U-test for non-paired samples. Time-related events such as freedom from explantation or other degeneration threshold values were evaluated according to Kaplan–Meier, including numbers at risk at 0, 5 and 10 years, as well as freedom-from-event rates at 0, 5 and 10 years, with their 95% confidence limits. Differences are described with pairwise probability value indications derived from the log-rank test, unless otherwise indicated. We are aware of the limitations of applying a Kaplan–Meier analysis to our data, which was developed for terminal, irreversible events such as death or explantation, and not for potentially reversible events, such as an occasionally higher gradient that might decrease by the next examination. To reflect the clinical reality in such cases, i.e. to illustrate the proportion of explanted, well-functioning and dysfunctional grafts at the various examination intervals, we modified the statistical techniques recommended by Akins *et al*. [14]. The same method was applied in other matched comparisons [10, 13].

Illustrations for the development of gradients or insufficiency over time were obtained by considering the first appearance of a peak gradient (moderate = 25 mmHg or more, severe = 50 mmHg or more) or at least moderate insufficiency as terminal events, with the risk of a certain margin of error that could occur by overestimating or underestimating the parameters. However, because this error would then occur in all three types of conduits assessed, and because large variations of these parameters in the course of a single patient are rare, we felt that this method was sufficient for the purposes of the analysis.

Pulmonary valve annulus measurements were converted to age and body size-dependent Z-scores for DPH and BJV patients, according to the approach proposed by W. Berdau (Medical University of Kiel, Germany, 1989). Interpolation lines (Lowess fit) were calculated for Z-value groups (−2; 0; 2;…) that included conduits with ±1 Z-value with respect to the indicated value (e.g. 0 = −1 to +1). Z-values of groups were calculated for the size at implantation and are displayed over time according echocardiographic measurements. The variation between size at implantation and first echocardiographic measurements explains offset differences in the respective groups.

A limited number of annulus diameters for CHs over the period of 10 years disabled the calculation of Z-value developments for CHs; data for BJVs were available from a large European trial [15].

SPSS 23 (IBM Corporation, Somer, NY) was used for the analyses; a probability value of 0.05 or less was considered statistically significant.

## RESULTS

A total of 131 DPHs were implanted for PVR in the period from January 2005 to September 2015. Of these, 36 DPHs have been implanted within a prospective European clinical trial on DPHs, which has been running since October 2014 (ClinicalTrials.gov NCT 02035540, www.espoir-clinicaltrial.eu) at seven clinical sites (Hannover, Chisinau, London, Leiden, Leuven, Padua and Zurich) and two DPHs were implanted in the framework of a clinical study in Osaka, Japan. These results will be published elsewhere.

**Figure 1:**
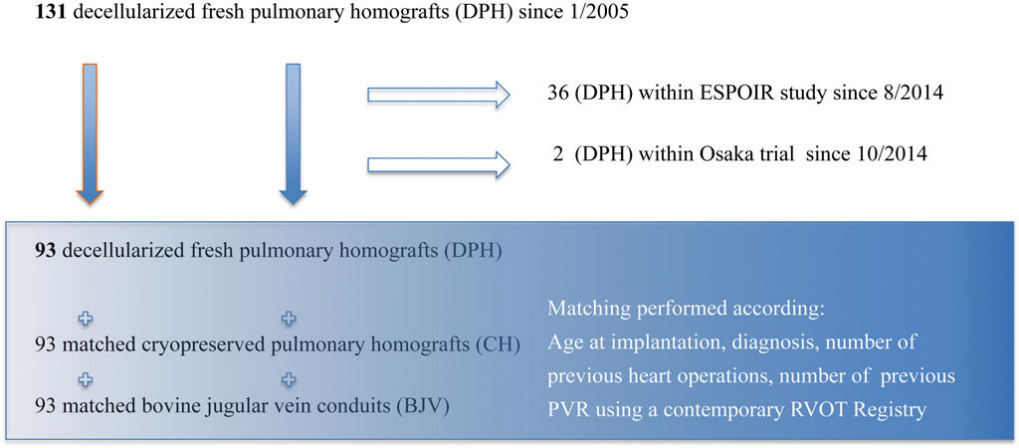
Study cohort overview.

Since January 2005, 93 DPHs have been implanted in Chisinau (*n* = 18) and in Hannover (*n* = 75). These 93 patients (58 males and 35 females) formed the study cohort for a matched comparison with CHs and BJVs (Fig. 1). Four DPH implantations were performed during combined aortic valve replacement and PVR for complex congenital heart disease.

The mean age at DPH implantation was 15.8 ± 10.21 years (CH 15.9 ± 10.4, BJV 15.6 ± 9.9 years), and the mean DPH diameter was 23.9 mm (CH 23.3 ± 3.6, BJV 19.9 ± 2.9 mm). There was 100% follow-up in DPHs, including 905 examinations with a mean follow-up of 4.59 ± 2.76 years, amounting to a total of 427.27 patient-years. Tetralogy-of-Fallot was the most frequent malformation (50.5%). Details of the three study groups are summarized in Table 1.

**Table 1:** Data of study groups

Implantation period	BJV	CH	DPH
	1999–2012	1985–2014	2005–2015
Diagnoses			
TOF	64	51	47
Ross	8	13	11
PI/PS	6	4	14
PA	6	8	9
DORV	5	9	5
TAC	2	2	2
TGA	1	4	4
Endocarditis	1	2	1
Total	93	93	93
Mean age at implantation [years]	15.6 ± 9.9	15.9 ± 10.4	15.8 ± 10.2
Mean follow-up [years]	6.4 ± 3.8	7.4 ± 5.8	4.6 ± 2.8
Total follow-up [years]	553.0	678.3	427.3
Sex (male)	41 (44%)	56 (60%)	58 (62%)
Number of previous operations			
0	11	19	16
1	50	40	51
2	22	27	14
>2	10	7	12
Type of previous procedures			
None	0	4	0
Homograft	13	23	12
Hancock valved conduit	1	5	6
Bovine jugular vein	10	1	7
Other valved conduit	3	0	2
Unvalved Dacron tube	2	1	3
Catheter-based intervention	10	7	23
Open valvulotomy	0	0	0
Extracardiac palliation, as BT shunt	14	12	6
Intracardiac repair, as RVOT patch	64	44	49
Other procedures	2	5	3
Conduit diameter [mean, mm]	19.9 ± 2.9	23.3 ± 3.6	23.9 ± 4.3
12–19 [mm]	28	11	14
20–23 [mm]	63	30	28
24–29 [mm]	2	52	51

**Figure 2:**
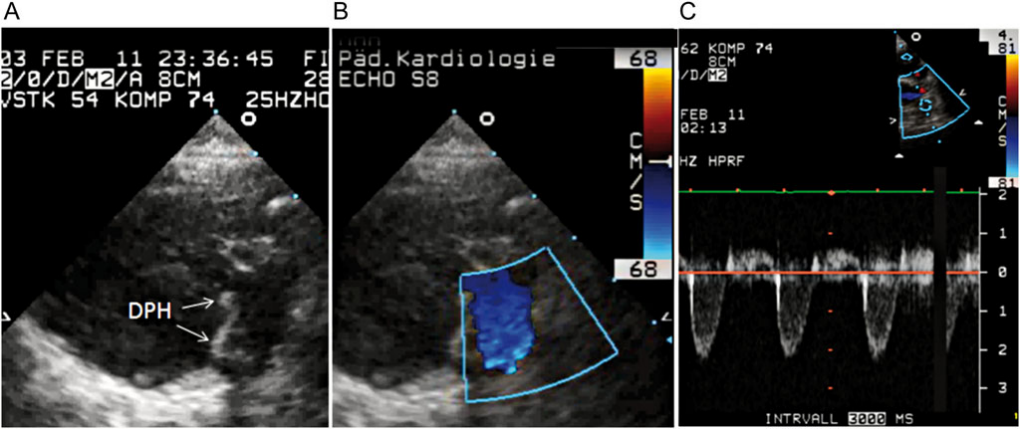
Postoperative echocardiography in a 16-week old girl, who died 3 months after DPH implantation due to sepsis. (**A**) 2-dimensional echocardiography in the short-axis view along the right ventricular outflow tract and DPH; (**B**) Colour-Doppler image at DPH in systole; (**C**) pulse-wave Doppler signal at DPH level showing laminar flow and mild regurgitation.

**Figure 3:**
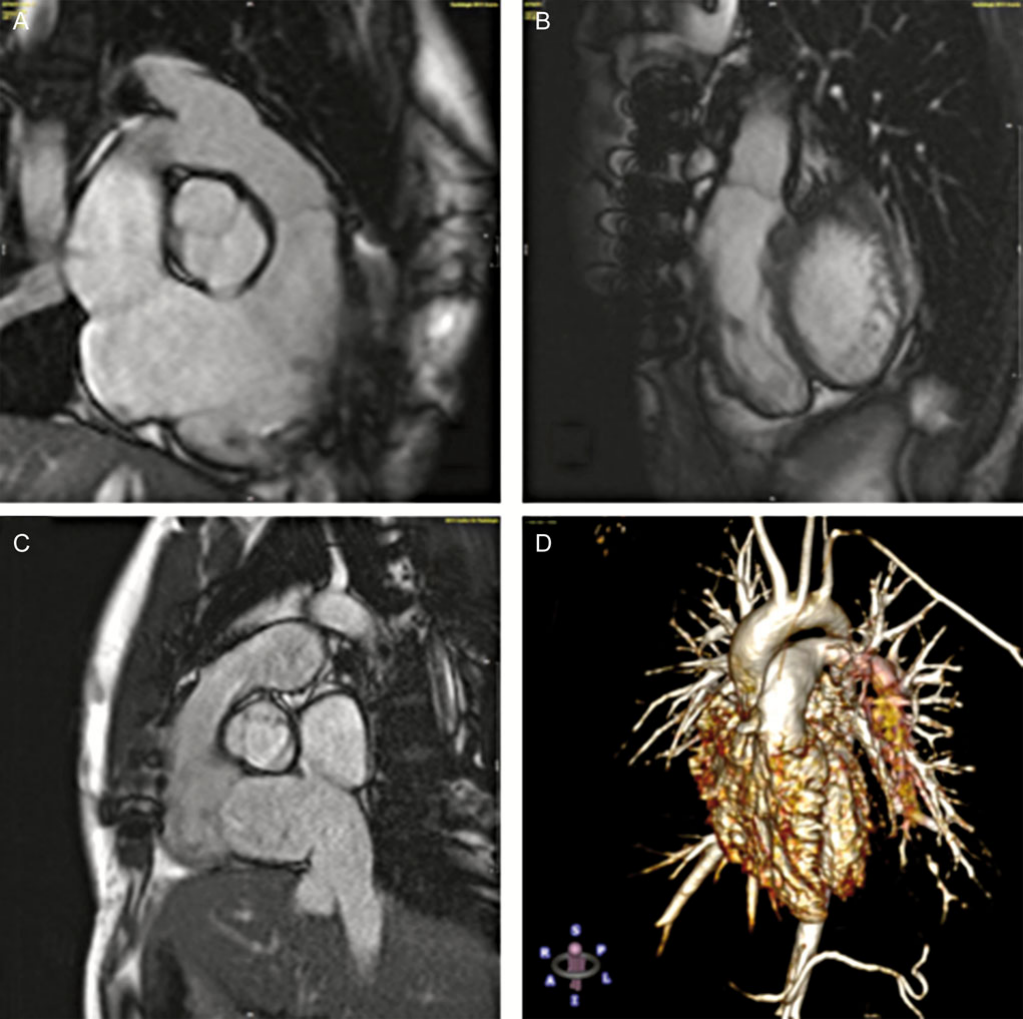
Cardiac magnetic resonance imaging examples of DPH. (**A**) Coronary three-chamber view at diastole 72 months after DPH implantation in a 20-year old patient; (**B**) sagittal view of the patient (A) at diastole; (**C**) sagittal view of DPH at systole 78 months after implantation in a 24-year old patient; (**D**) contrast-enhanced angiography 116 months after DPH implantation in a 10-year old patient.

In total, there have been two deaths in DPH patients, both valve-unrelated. One 18-year old patient died in Moldova 11 months postoperatively due to a suspected mesenteric infarction resulting from cardiac arrhythmia. The intact valve was explanted and histological results published [10]. The other patient was a 6-week old girl with absent pulmonary valve syndrome who died 10 weeks postoperatively at Hannover Medical School due to sepsis caused by persisting postoperative chylothorax with a drainage volume of up to 1 l daily. The valve showed no signs of endocarditis on echocardiography and good function with mild regurgitation and no stenosis (Fig. 2). Parental consent for an autopsy was denied. Figure 3 provides mid-to longterm CMR imaging examples of DPH.

### Intraoperative handling

PVR with DPH has proved to be safe. One patient at Hannover Medical School required re-operation (1.1%) on the same day due to a haematoma leading to graft compression. Aside from this case, no other perioperative complications such as DPH rupture or dislodgement were observed.

### DPH performance in comparison with CH and BJV

**Figure 4:**
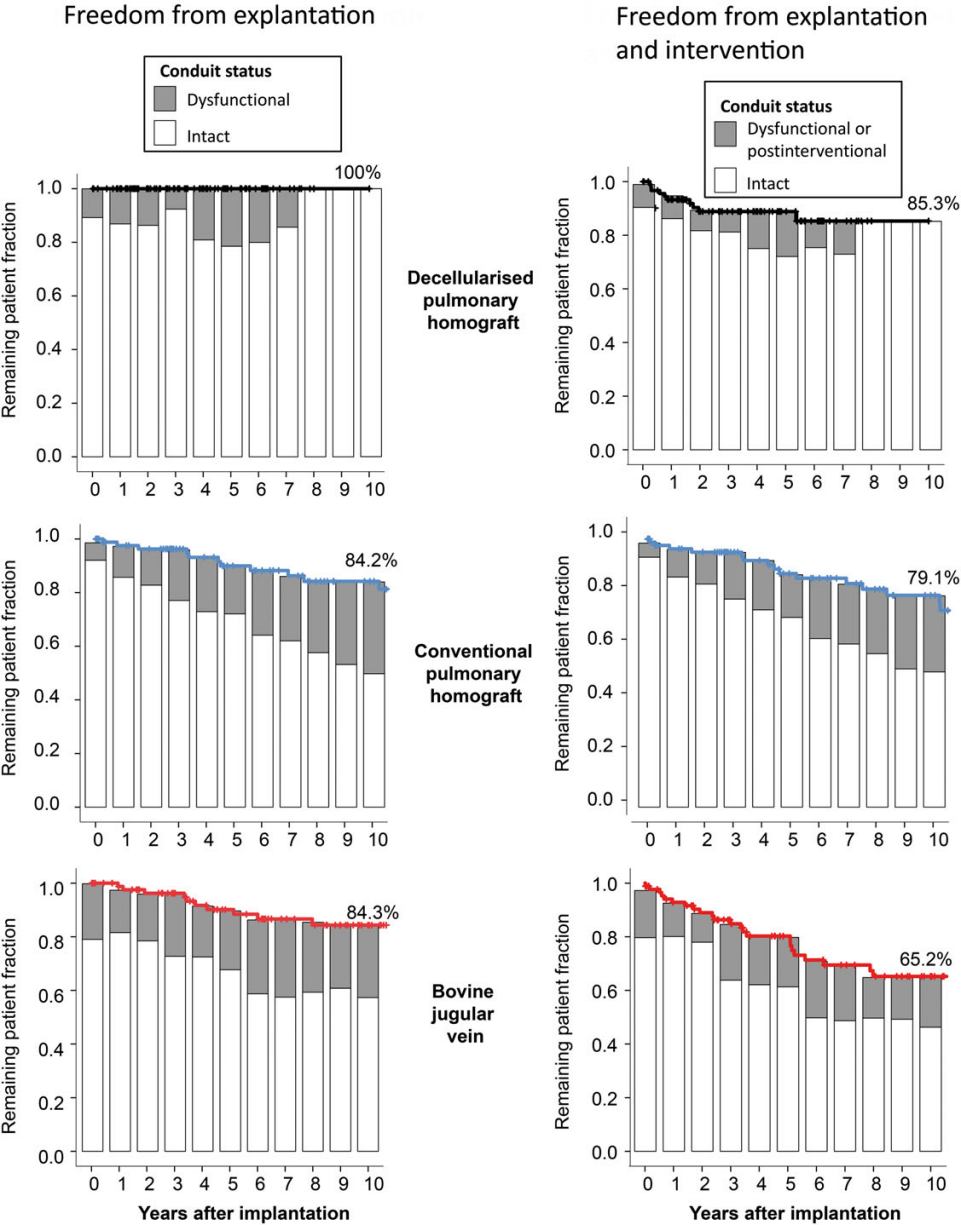
Freedom from explantation and freedom from explantation/catheter intervention, including percentage of conduits with degeneration signs for DPH, CH and BJV.

Figure 4 shows freedom from explantation and the number of DPH grafts (in dark grey) with degeneration signs in comparison with CH (in blue) and BJV (in red) in the left column and freedom from explantation and/or catheter intervention in the right column. At 10 years, the rate of freedom of explantation was 100% for DPH, 84.2% for CH (*P*= 0.01 vs DPH) and 84.3% for BJV (*P* = 0.01 vs DPH).

All CH and BJV explants were performed due to haemodynamic relevant degeneration and not on the occasion of another procedure.

**Figure 5:**
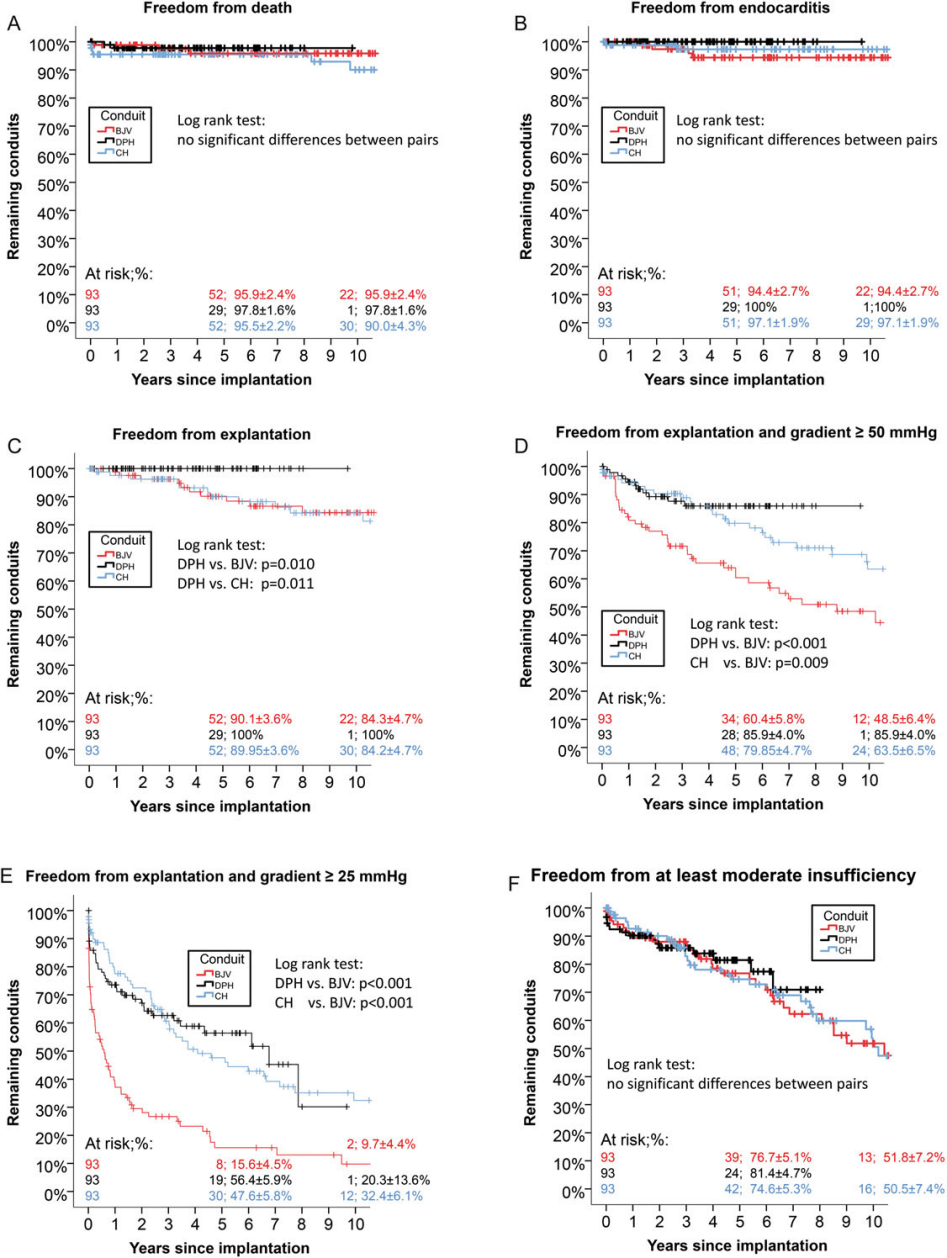
Freedom from death (**A**), freedom from infective endocarditis (**B**), freedom from explantation (**C**), freedom from explantation and trans-conduit gradients ≥50 mmHg (**D**), freedom from explantation and trans-conduit gradients ≥25 mmHg (**E**), freedom from at least moderate insufficiency (**F**) for DPH, CH and BJV as Kaplan–Meier curves.

At 10 years, the rate of freedom of death was 98% for DPH, 90% within the matched CH patients (n.s.) and 96% within the matched BJV patients (n.s.). At 10 years, freedom from infective endocarditis (IE) was 100% for DPH, 97.3 ± 1.9% within the matched CH patients (*P* = 0.2 vs DPH) and 94.3 ± 2.8% for BJV patients (*P* = 0.06 vs DPH). For details, see Fig. 5A and B. Figure 5C shows the Kaplan–Meier curve for freedom from explantation; Fig. 5D shows freedom from explantation and a trans-conduit ≥50 mmHg and Fig. 5E shows freedom from explantation and a trans-conduit ≥25 mmHg with the respective log-rank test results. Freedom from at least moderate valvular regurgitation was not statistically different between DPH, CH and BJV (Figure 5F).

**Figure 6:**
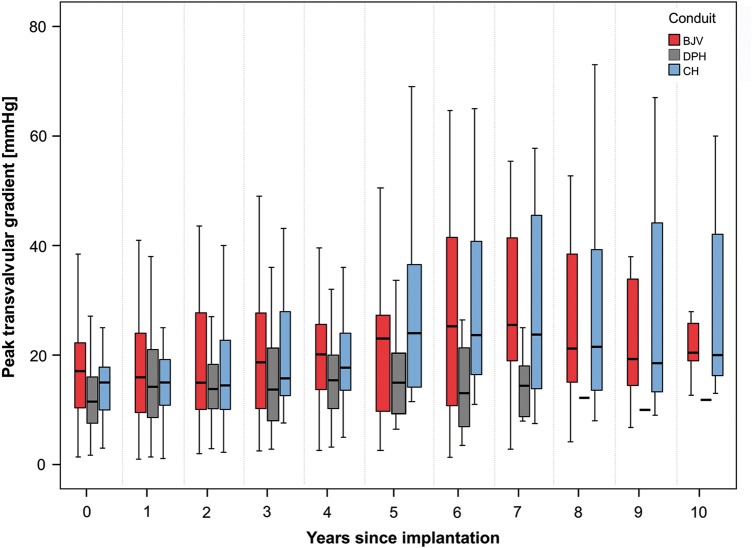
Peak valvular gradient development for DPH, CH and BJV within 10 years after implantation.

Figure 6 shows the echocardiographic peak valvular gradients in mmHg for DPHs, CHs and BJVs throughout the 10-year observation period, with the lowest gradients observed for DPHs.

### Annulus size development

**Figure 7:**
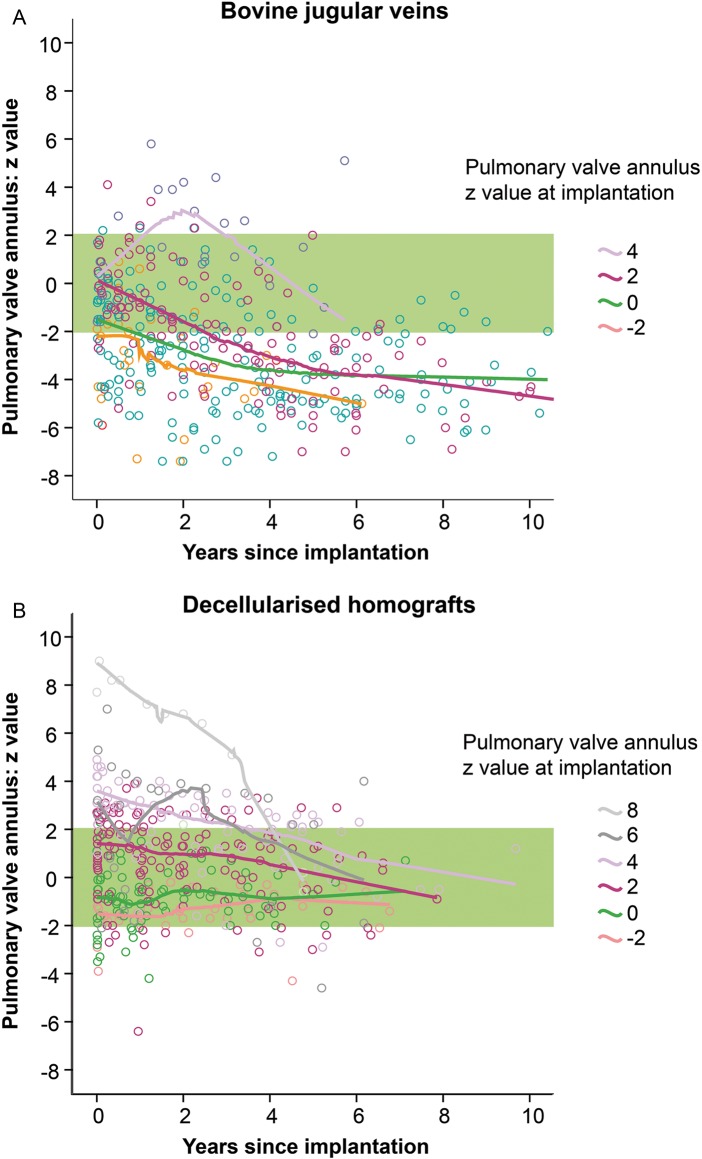
*Z*-Score development of pulmonary valve annulus for BJV (**A**) and DPH (**B**) within 10 years after implantation.

In Fig. 7, the development of pulmonary annulus diameter is shown for DPHs and BJVs as age and weight-reflecting Z-scores over the 10-year follow-up period, differentiated by the conduit size at implantation. Notably, there were more oversized implantations in DPHs. However, all BJVs implanted with physiological sizes (−2 Z-score to Z-score + 2) fell below the normal range, whereas DPH Z-scores stayed within the normal range, regardless whether they were oversized, regular-sized or undersized at implantation.

## DISCUSSION

A comparative study of any option for PVR is hampered by the diversity of the patients treated. Young patients are far more likely to exhibit accelerated degeneration of the biological grafts used in this age group [13, 16, 17]. More complex congenital malformations will lead to earlier degeneration of the implanted RVOT graft than implantations in the almost ideal situation of a Ross patient, which, in addition, is normally carried out in adult patients [18]. Even in a medium complex congenital malformation such as Tetralogy of Fallot, there is significant variability between centres in terms of timing and the type of PVR procedures. As the annual rate of PVR is increasing rapidly with a corresponding impact on survival [19, 20], it is important to reasonably estimate the best approach.

We have chosen a matched comparison with the two most widely used grafts for paediatric PVR -BJV and CH- to evaluate the mid-term results of DPHs. This matching was based on the factors described above and therefore allowed a valid comparison of the results. DPHs showed superior results to CHs and BJVs with respect to freedom from degeneration and subsequently freedom from explantation. However, a number of DPH grafts nevertheless exhibited stenosis and/or regurgitation within the study period. This was caused predominantly by distal anastomosis stenosis, which is not an unusual diagnosis in patients with multiple pulmonary artery procedures. This is an important finding as it demonstrates the susceptibility of DPHs to such problems and underlines the importance of adequate surgical implantation for decellularized grafts, which, due their softness, are also prone to valvular regurgitation when implanted suboptimally. Within the 10-year period, valvular gradients, however, did not increase with DPHs, a result that was not achieved by CHs and BJVs.

Two other groups have also reported on their results using DPHs for PVR and have demonstrated superior results to those obtained using conventional cryopreserved homografts [21, 22]. Even though the decellularization protocols used differ to our technique, this can nonetheless be seen as another indicator of the excellent results achieved using decellularized allografts. In view of these results, the question may arise in the future whether allograft cryopreservation should not be superseded by decellularization techniques as the current gold standard.

IE is a severe threat for any heart valve prosthesis. Although endocarditis of the pulmonary valve is rarer than in left-sided valves, the impact on the patient can be substantial. CHs, when used for PVR, have demonstrated a rate of freedom from IE of 97.3% at 10 years in a recent comparison with BJVs [23]. We observed identical results within our CH cohort. BJVs, which have shown dramatic rates of IE of up to 20% [24], in our matched comparison also exhibited the highest rate of IE, whereas no IE was observed with DPHs in a total follow-up of over 400 patient-years. Although this finding failed to reach statistical significance, it seems to be worthy of careful monitoring in the future. We have shown reduced antigenicity in DPHs on cellular and humoral levels, which could translate to less susceptibility to bacterial adhesion [11, 12]. We implanted DPH in one patient after completion of antibiotic treatment for IE in an infected RVOT conduit and observed no recurrence of IE. Nevertheless, we recommend following the current guidelines for IE prophylaxis and would not recommend the use of DPHs in active IE.

DPHs showed, in contrast to BJVs, normal Z-values over a period of up to 10 years. Growth potential is one of the unique features of DPHs and our mid-term results confirm the adaptive growth described in earlier reports [10]. However, growth potential depends on recellularization by metabolically active recipient's cells. Using a growing sheep model, the Padua group has shown that recellularization predominantly occurs from the adventitial side in decellularized allografts [25].

However, there are no data, either clinical or preclinical, which provide an insight in the amount of recellularization in re-do settings after one or multiple thoracotomies. It seems apparent that rapid and thorough recellularization is less likely in the setting of severe scarring around the graft. Laminar flow across the outflow tract is a further prerequisite for sufficient colonization, as recellularization from the luminal side is also important, e.g. for cusps. Mechanical alteration, e.g. due to moderate graft distortion, not only may impede immediate valvular competence of the soft cusps of decellularized homografts, but also is important for long-term function and growth potential, as long-term turbulence will have adverse effects even on native semilunar valves. Future analysis will need to assess the growth potential of DPHs with respect to such factors on the basis of more implantations.

### Limitations

Findings are necessarily limited by the number of DPHs implanted so far. However, prospective European-wide multicentre trials on the use of DPHs for pulmonary and aortic valve replacement have been initiated, which will provide further data, and the follow-up of DPH patients is ongoing.

Limitations are also present by the matching process itself, which will not allow a 100% perfect matching of patients given the low numbers and variance in congenital heart disease. Within the RVOT Registry, there is slight imbalance regarding the implantation periods of DPHs and CHs, which is explained by the preferential use of DPHs in recent years at our institution and the limited number of available homografts. As there have been no new milestone achievements in allograft cryopreservation within the past years and as CH results are in line with the literature, we do not consider this to be a significant limitation.

Data within the Registry originates from 7 experienced international centres, which may introduce bias by different re-operation algorithms. On the other hand, the Registry is minimizing centre effects by its unique number of available RVOT conduits.

The lack of histological data resulting from the lack of DPH explantations to date, albeit in itself a positive result, presents a limitation for the evaluation of the recellularization potential of DPHs in humans.

## CONCLUSION

Mid-term outcomes of decellularized fresh pulmonary homografts used for PVR confirm earlier results of reduced re-operation rates compared with the current gold standard CH and BJVs. For future investigations, more data, especially on smaller grafts and recellularization conditions, are required, together with long-term performance monitoring, to conduct a more detailed evaluation.

## Funding

This study was supported by a grant from the European Union's Seventh Framework Programme for Research, Technological Development and Demonstration under Grant Agreement No. 278453. Funding to pay the Open Access publication charges for this article was provided by the European Union's Seventh Framework Programme for Research, Technological Development and Demonstration under Grant Agreement No. 278453.

**Conflict of interest:** A. Haverich holds shares in corlife oHG, a company for the future processing of decellularized allografts, equivalent to those used in this study.

## APPENDIX. CONFERENCE DISCUSSION

***Dr L. Galletti*** *(Bergamo, Italy):* The Hannover Group has been promoting for years the use of decellularized fresh homografts, actually being a recognized leader in using this technique. This process allows for a very good function of the homografts in the mid and long-term follow-up.

Using the statistical method of matched analysis that allows comparison between each individual case, decellularized homografts performed better than non-decellularized homografts and bovine jugular vein conduits.

However, in this experience, the vast majority of the tube was put in older patients, to restore pulmonary valve competence after previous tetralogy repair, and could be a favorable subset of patients.

Now in the literature, as well as in the clinical experience of most centers, factors associated with the failure of the valve and the conduit are mainly three: Age at implant; size of the conduit; and the anatomic versus non-anatomic position, or if you prefer use in Ross versus non-Ross procedure.

So my first question is, based on your data and your experience, do you think that the use of the decellularization process could neutralize those risk factors and prolong the life of the conduit even in a less favorable situation?

***Dr Sarikouch:*** Dr Galletti, thank you for the question. No, I do not think that decellularized homografts will eliminate all these risk factors. Complex surgical procedure as a second or third redo in a small child would stay a complex surgical procedure, and may eventually lead to supravalvular stenosis, whether you use a decellularized homograft or a standard cryopreserved homograft, or a Contegra.

We think that the recellularization will depend on the lamina flow, and normal valve stress once implanted. So this will for sure also influence the long-term results in decellularized homografts.

***Dr Galletti:*** But anyway, in your data, there is some suggestion of a potential growth of this conduit that could be a good thing in a younger patient?

***Dr Sarikouch:*** Yes. So as you have noticed, we have called this adaptive growth. We are cautious. Only 15% of the homografts implanted are below 20 mm of size. So that is not a huge amount of data for small sizes. However, that is ten years of follow-up, and we did not see one decellularized homograft out of the normal range, and we did not observe this phenomenon in the other valve substitute in that period.

***Dr Galletti:*** Okay, the second question is related to the protocol *per se*. Can you maybe give us some information if you think this technique is reproducible by other homograft banks? Also finally, what could be, if you know, the extra cost compared to the regular or standard homograft that we regularly use in the clinical practice?

***Dr Sarikouch:*** The process of decellularization is completely standardised. It is validated and it is quality controlled. Otherwise, it would not have been approved by the authorities in Germany and adopted by the authorities in Switzerland, Netherlands, Italy, United Kingdom and Belgium. So it is reproducible.

Regarding the costs, so the costs are high, and they are in the range of the Melody valve during introduction into the market. However, the price will decrease as more and more homograft banks will use this technique.

Also, as most of the costs are related to standardisation and quality controls, clean room facilities, etc. I do not think that every homograft bank has to establish such a process. Maybe one per nation is enough, or maybe establish trans-national collaboration for this technique, as we have done for our European study, as far as where we want to analyze 200 pulmonary valve replacements in a prospective manner.
